# Molecular Analysis of the Notch Repressor-Complex in *Drosophila*: Characterization of Potential Hairless Binding Sites on Suppressor of Hairless

**DOI:** 10.1371/journal.pone.0027986

**Published:** 2011-11-18

**Authors:** Patricia Kurth, Anette Preiss, Rhett A. Kovall, Dieter Maier

**Affiliations:** 1 Institut für Genetik (240), Universität Hohenheim, Stuttgart, Germany; 2 Department of Molecular Genetics, Biochemistry and Microbiology, University of Cincinnati College of Medicine, Cincinnati, Ohio, United States of America; Instituto Nacional de Câncer, Brazil

## Abstract

The Notch signalling pathway mediates cell-cell communication in a wide variety of organisms. The major components, as well as the basic mechanisms of Notch signal transduction, are remarkably well conserved amongst vertebrates and invertebrates. Notch signalling results in transcriptional activation of Notch target genes, which is mediated by an activator complex composed of the DNA binding protein CSL, the intracellular domain of the Notch receptor, and the transcriptional coactivator Mastermind. In the absence of active signalling, CSL represses transcription from Notch target genes by the recruitment of corepressors. The Notch activator complex is extremely well conserved and has been studied in great detail. However, Notch repressor complexes are far less understood. In *Drosophila melanogaster*, the CSL protein is termed Suppressor of Hairless [Su(H)]. Su(H) functions as a transcriptional repressor by binding Hairless, the major antagonist of Notch signalling in *Drosophila*, which in turn recruits two general corepressors – Groucho and C-terminal binding protein CtBP. Recently, we determined that the C-terminal domain (CTD) of Su(H) binds Hairless and identified a single site in Hairless, which is essential for contacting Su(H). Here we present additional biochemical and *in vivo* studies aimed at mapping the residues in Su(H) that contact Hairless. Focusing on surface exposed residues in the CTD, we identified two sites that affect Hairless binding in biochemical assays. Mutation of these sites neither affects binding to DNA nor to Notch. Subsequently, these Su(H) mutants were found to function normally in cellular and *in* vivo assays using transgenic flies. However, these experiments rely on Su(H) overexpression, which does not allow for detection of quantitative or subtle differences in activity. We discuss the implications of our results.

## Introduction

The Notch signalling pathway is highly conserved in metazoans, where it allows for intercellular communication during the specification of cell fates [1]. *Notch* encodes a single pass transmembrane receptor that is activated by transmembrane ligands presented by the signalling cell. As consequence of receptor activation, the intracellular Notch domain (ICN) is cleaved and migrates to the nucleus. There it binds to the CSL-type DNA-binding protein (C-promoter binding factor 1 [CBF-1] in *H. sapiens*, [lag-1] in *C. elegans*, Suppressor of Hairless in *D. melanogaster* [Su(H)]), and assembles, together with the coactivator Mastermind (Mam), a transcriptional activator complex (overview in: [1]–[4]. Formation of the CSL-ICN-Mam ternary complex, in conjunction with other transcriptional components, results in the activation of Notch target genes, *e.g.* the *Hairy* and *Enhancer of split* (HES) family of genes. HES genes encode transcriptional repressors that function to shut down gene expression for genes that confer the primary cell fate, thereby enforcing a secondary fate within the signal-receiving cell [1]–[2].

The components of the activator complex (CSL-ICN-Mam) are highly conserved from worms and flies to humans in both primary sequence and the overall three-dimensional structure of this complex [5]–[6]. The central molecule of the activator complex is CSL, which contains three functional domains: the N-terminal domain (NTD), beta–trefoil domain (BTD), and C-terminal domain (CTD). Both the NTD and BTD contact DNA. The BTD and the CTD interact with ICN, whereby BTD forms a high-affinity interaction with the RAM domain of ICN and the CTD binds both the ankyrin repeats (ANK) of ICN and Mam [5]–[6], overview in [3].

In the absence of signal, CSL interacts with transcriptional corepressors to turn off transcription from Notch target genes. Similar to the activator complex, CSL is the central component of the repressor complex; however, in contrast to the activator complex, the structure of the repressor complex is still unknown. Human CBF-1 has been shown to interact with several different corepressors, *e.g.* SMRT/NCOR, MINT/SHARP, KyoT2, and CIR. Most of these corepressors contact a site within the BTD of CBF-1 that likely overlaps where the RAM domain of Notch binds. This has led to a model, in which the repression and activation of Notch target genes is mediated by the competition of ICN and corepressors for binding CBF-1 (overview in [7]). In *D. melanogaster*, the transcriptional corepressor Hairless is the major antagonist of Notch signalling (reviewed in [8]). Hairless binds to the CTD of Su(H) – the fly CSL protein - and recruits two additional corepressors, the C-terminal binding protein (CtBP) and Groucho (Gro). Together this repressor complex silences expression from Notch target genes ([9]–[12]). Hence, CSL plays a dual role in both activator and repressor complexes.

We have initiated a detailed analysis of the Notch repressor complex in *Drosophila*. Recently, we have shown that Hairless and Su(H) form a high affinity complex, and that mutations within Su(H) that affect binding of ICN have no effect on Hairless binding. Nonetheless, Hairless and Notch compete for Su(H) in vitro, despite the disparities in affinities of ICN and Hairless for the CTD of Su(H). Moreover, we have mapped a single residue in Hairless that is crucial for binding Su(H) ([10]). To further our understanding of Notch signalling and the repressor complex in *Drosophila*, we have analysed 17 single, double, and triple mutations in the CTD of Su(H) for their involvement in the binding of Hairless using a yeast two-hybrid assay. A double mutation was identified that strongly reduces interactions with Hairless, but neither affected DNA nor Notch binding by Su(H). In spite of this reduction in binding, overexpression of the Su(H) double mutant in a transcriptional cell culture assay, as well as in the fly, revealed little to no changes in function compared to wild type Su(H). These results were unexpected and we consider two possibilities: (1) potentially other residues in Su(H) contribute to the binding of Hairless, which allows for a sufficiently strong interaction *in vivo*; or (2) alternatively, the presence of endogenous Su(H) in our cellular and *in vivo* assays distorts our results.

## Results

### Identification of potential Hairless binding sites in the CTD of Su(H)

Recently, we have identified the C-terminal domain of Su(H) as the binding domain for Hairless (CTD, amino acids 417–528). Binding to Hairless was enhanced by the presence of the N-terminal α-helix (amino acids 1–119), which helps to stabilize the folding of the CTD. Mutations that affect binding to ICN did not interfere with the binding to Hairless, suggesting that ICN and Hairless do not compete for the same contact sites in Su(H) CTD [10].

**Figure 1:**
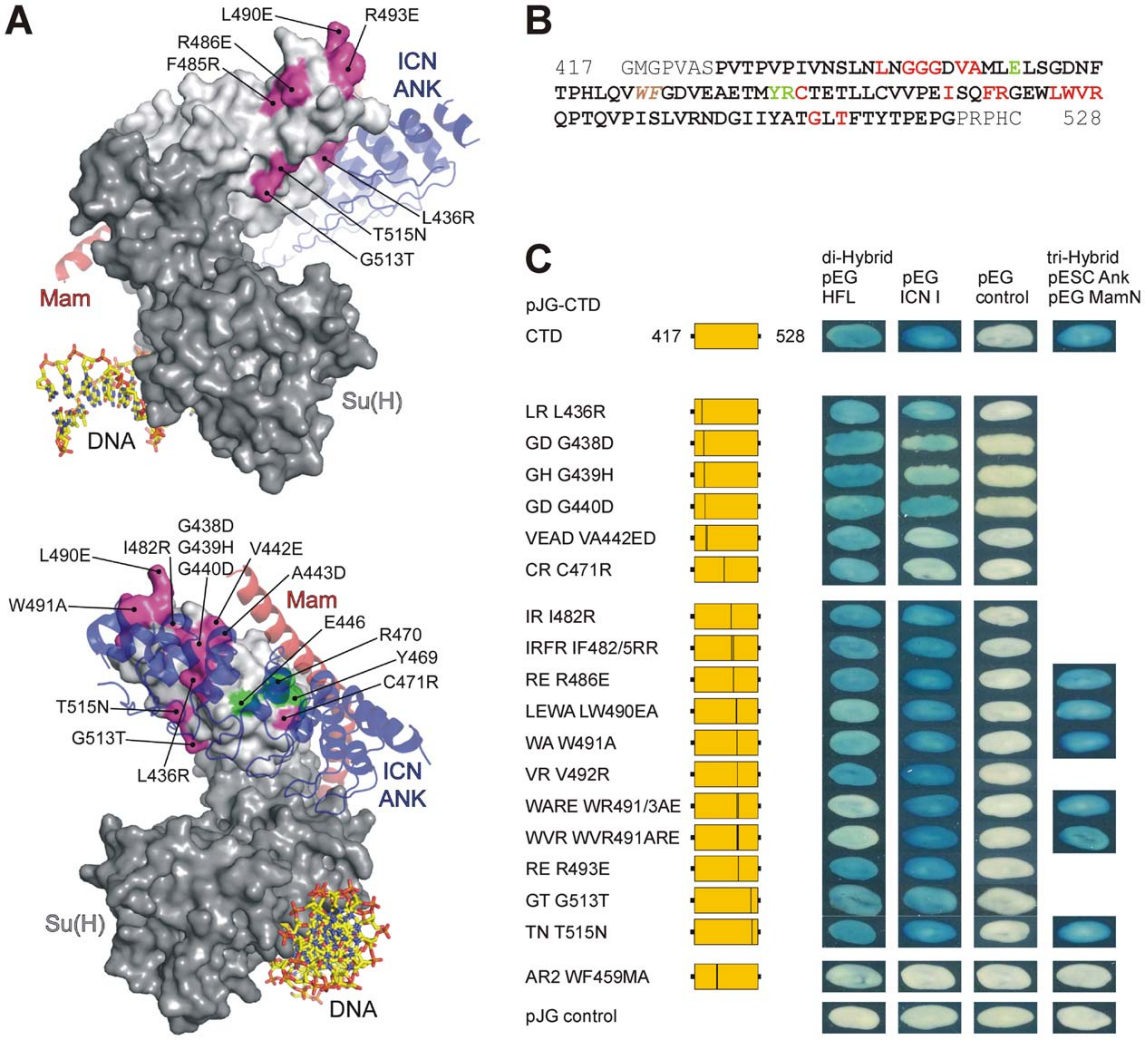
Fine mapping of the Hairless contact sites on Su(H) CTD. A) Surface representation of CSL-DNA structure with the NTD, BTD, and CTD coloured in dark and light grey, respectively. The DNA is in a stick representation with carbon, oxygen, nitrogen, and phosphorous atoms coloured yellow, red, blue, and orange, respectively. Notch ANK is coloured in blue and MamN in red, and represented as transparent ribbons. The residues on the CTD that interacted with ICN and Mam in the yeast two-hybrid assay are coloured green. Residues that were mutated in the course of this work are coloured magenta. B) Primary sequence of the Su(H)-CTD construct; the CTD is shown in bold. Amino acids shown to contact ANK/Mam are depicted in green; red are those tested for Hairless binding, and brown depicts the AR2 mutation that disrupts the CTD fold. C) Mutant CTD constructs were tested in a yeast two-hybrid assay for binding to full length Hairless (HFL) and to intracellular Notch (ICN I). Moreover, the mutant CTD constructs were tested in a yeast three-hybrid assay for their potential to assemble the ternary activator complex with ANK and MamN. Empty vectors served as negative controls. Relative position of mutations within the CTD is indicated. Note that binding of Hairless but not of ICN I to LEWA or WA is reduced and is nearly abolished in WARE and WVR. However, all these mutants display normal binding to Notch and are capable of forming a ternary activator complex. The constructs GD, GH, VEAD and CR bear mutations at the CTD-ANK and CTD-Mam interfaces, consistent with a strongly reduced binding to ICN I. Hence, Notch and Hairless contact different sites on Su(H). Mutant AR2, in which residues within the hydrophobic core of CTD are mutated, is likely compromised for folding, and fails to bind either HFL or ICN I.

To identify the amino acids in CTD responsible for interaction with Hairless, a total of 17 single, double or triple amino acid substitutions were introduced by *in vitro* mutagenesis. The main criterion for the changes was (1) whether the amino acids were surface exposed, which was based on the orthologous mammalian and *C. elegans* CSL structures; and/or (2) within a putative protein-protein interaction domain that was determined computationally (http://sppider.cchmc.org) (Fig. 1A,B). The sites of mutation were changed to residues that would likely interfere with Hairless binding (Fig. 1B,C). The mutant constructs were tested in a yeast two-hybrid assay using Hairless or ICN I as bait (Fig. 1 C). In addition, we assayed for the formation of the ternary activator complex consisting of Su(H), Notch Ank and MamN (Fig. 1C; [10]). For the majority of mutants examined no changes in binding were detected. However, four mutations showed reductions in Hairless binding: CTD^LEWA^ (L490E/W491A), CTD^WA^ (W491A), CTD^WARE^ (W491A/R493E) and CTD^WVR^ (W491A/V492R/R493E). Together, this assay revealed that the residues Tryptophan 491 and Arginine 493 are likely important for the binding of Hairless, because the combined mutation CTD^WARE^ nearly abolished Hairless binding in the yeast assay. In addition, Leucine 490 appeared to contribute since CTD^LEWA^ bound less well than CTD^WA^, whereas mutation of Valine 492 did not further reduce binding in CTD^WVR^. The single mutations CTD^RE^ (R493E) and CTD^VR^ (V492R) were without effect (Fig. 1C).

Two mutations CTD^VEAD^ (V442E/A443D) and CTD^CR^ (C471R) nearly abolished binding to ICN I, and two other mutations CTD^GD^ (G438D) and CTD^GH^ (G439H CTD^CR^) reduced binding to ICN I; however, none of these mutations affected binding to Hairless (Fig. 1C). CTD^CR^, CTD^GD^, and CTD^GH^ lie within the region known to contact the Notch Ankyrin repeats, whereas CTD^VEAD^ is in the vicinity of ANK and MAM, but does not make direct contact with these proteins. Two controls were included, the empty vector and the double mutant CTD^AR2^ (W459A/F460A). The CTD^AR2^ mutant affects amino acids buried within the hydrophobic core of CTD and is hence predicted to disrupt CTD folding. As expected, both controls did not bind to either Hairless or ICN I, and failed to assemble the ternary complex with N-Ank and MamN (Fig. 1C).

**Figure 2:**
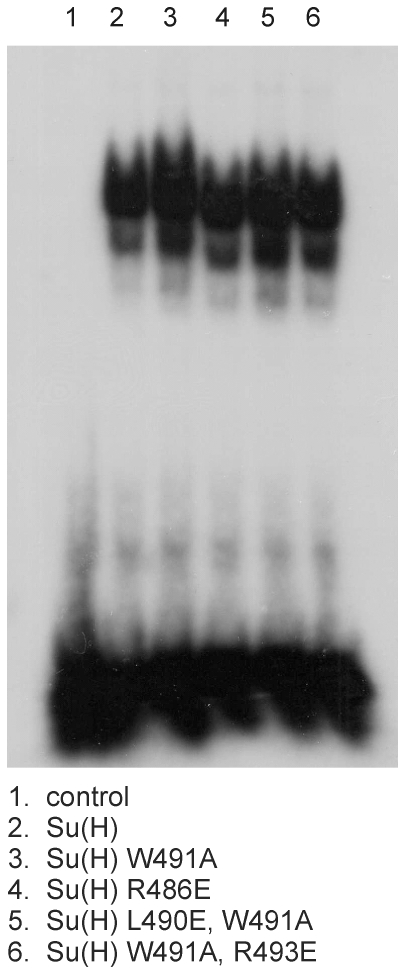
DNA binding is not altered in Su(H) mutants. Electromobility shift assay for binding of Su(H) protein variants to the radiolabelled *E(spl)*m8-S1 oligo [44]. Control, no protein added (lane 1). The binding of the mutant Su(H)^WA^, Su(H)^RE^, Su(H)^LEWA^, or Su(H)^WARE^ proteins (lane 3–6) to DNA was similar as the wild-type Su(H) protein (lane 2).

Based on the structures of mammalian and worm CSL proteins, Su(H) is expected to bind DNA with its N-terminal and beta-trefoil domains (NTD, BTD; Fig. 1A). Accordingly, mutations in CTD should not interfere with DNA binding, which was confirmed by an electrophoretic mobility shift assay (EMSA) with the relevant Su(H) mutants (Fig. 2). To this end, R486E, W491A, L490E/W491A and W491A/R493E mutations were introduced into full length Su(H) cDNA that was *in vitro* transcribed and translated. The *E(spl)* m8 oligo-nucleotide containing a Su(H) binding site (m8-S1, [44]) was used as the target DNA. No difference in DNA-binding was observed between wild type and mutant Su(H) protein (Fig. 2).

### The CTD^WARE^ double mutation fails to bind truncated forms of Hairless

**Figure 3:**
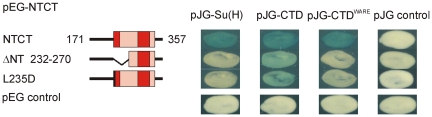
Su(H)^WARE^ binding capacity to Hairless mutants. Yeast two-hybrid assay to test for binding activity of wild type Su(H), CTD and mutant CTD^WARE^ constructs with Hairless NTCT, NTCT^ΔNT^ Dand NTCT^LD^; empty vector served as a negative control. Note the lack of binding of CTD^WARE^ with the ΔNT deletion or the NTCT^LD^ mutation.

Based on the near complete loss of binding to full length Hairless, the double mutation CTD^WARE^ (W491A/R493E) was chosen for further analysis. Previously, we defined a subdomain of Hairless, termed NTCT (amino acids 171–375; [10]), which recapitulated all of the binding of Hairless to Su(H) *in vitro*. Unexpectedly, we found that CTD^WARE^ bound to NTCT similarly to full length Su(H) and only slightly weaker than wild type CTD (Fig. 3). In addition, we tested the ability of two Hairless NTCT mutants, the NT-deletion NTCT^ΔNT^ and the single site mutant NTCT^LD^ (L235D) for binding to the Su(H) constructs. Both NTCT mutants fail to bind to full length Su(H) and showed a markedly reduced binding activity towards CTD (Fig. 3; [10]). However, NTCT^ΔNT^ and notably NTCT^LD^ completely failed to bind to CTD^WARE^, strongly indicating that the affected amino acids are involved in the binding of Su(H) and Hairless (Fig. 3).

### Su(H)^WARE^ gives a normal response in a transcriptional assay

**Figure 4:**
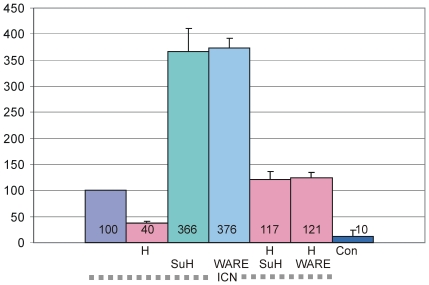
Activation and repression of a Notch reporter gene by Su(H) variants. Effects of the mutant Su(H)^WARE^ (WARE) on Notch ICN mediated expression from the NRE-reporter (luciferase reporter containing wild type Su(H) binding sites, [13]) were analysed in *Drosophila* S2 cell culture. Indicated constructs, Notch ICN, full length Hairless (H), full length Suppressor of Hairless (SuH) or the mutant Su(H)^WARE^ (WARE) were used to transiently transfect S2 cells; empty vector served as a negative control (Con). Luciferase activity is represented on the *y*-axis and transfection efficiency was normalized by cotransfection of the renilla plasmid. Values for NRE expression in the presence of Notch ICN were normalized to 100% (lane 1). The results confirm published data [10].

Thus far, our data indicated that the W491A/R493E amino acid substitutions had an effect on the Su(H)-Hairless interaction. In order to test the effect of this mutation in a more physiological setting, it was introduced into the Su(H) full length cDNA [Su(H)^WARE^] and transiently expressed in S2 cells. Subsequently, we analysed the transcriptional activation and repression of a luciferase reporter construct bearing Su(H) binding sites (NRE-reporter; [13]). We reasoned that Su(H)^WARE^ should behave as a transcriptional activator together with ICN, similar to wild type Su(H), because binding of Notch was unaffected by the mutation. This was indeed observed – transfection of ICN alone strongly activates the NRE-reporter, via endogenous Su(H), which was taken as 100% to normalize the other results [13]. Addition of the wild type Su(H) construct resulted in about three- to four-fold increase of luciferase activity (Fig. 4), which is in agreement with earlier observations [10], [14]. A likewise increase in reporter activity was obtained by adding Su(H)^WARE^, indicating that the mutant protein can efficiently assemble an activator complex in S2 cells (Fig. 4).

Assembly of the repressor complex was tested by cotransfecting the S2 cells with Hairless and ICN. This caused a strong downregulation of ICN mediated transcriptional activation of the NRE-reporter to about 40% [10], [12], because Hairless can assemble a repressor complex with endogenous Su(H) on the NRE promoter. Interestingly, Hairless is able to abrogate the strong activation mediated by the addition of exogenous Su(H) to near completion (Fig. 4; [10]), indicating that Hairless and Su(H) interact with each other. To our surprise, the same degree of repression was observed with Su(H)^WARE^ (Fig. 4). Apparently, Hairless binds the Su(H) mutant with sufficiently enough affinity to repress transcription as efficiently as the wild type Su(H) protein. This result was unexpected since the yeast two-hybrid data suggested a near complete lack of binding of Su(H)^WARE^ to Hairless. However, at this stage of our analysis, it was unclear whether this effect was specific to S2 cell culture.

### In vivo transcriptional response of Notch target genes during wing development

To analyse the *in vivo* activity of the mutant Su(H) protein, transgenic flies were established using the PhiC31 method [15]. This system avoids position effects and hence allows the direct comparison of different transgenes at the same location. Su(H)^WARE^ was cloned into an appropriate UAS-vector and integrated at the 96E landing site for comparison with the accordant Su(H) construct [10]. Moreover, the transgenic Su(H)^WARE^ line was recombined with full length Hairless HFL and with mutant Hairless H^LD^, each integrated at 68E, to allow for a combined overexpression. The latter completely failed to bind wild type Su(H) [10]. The wild type and mutant Su(H) and Hairless transgenes were locally overexpressed using the Gal4/UAS-system [16].

**Figure 5:**
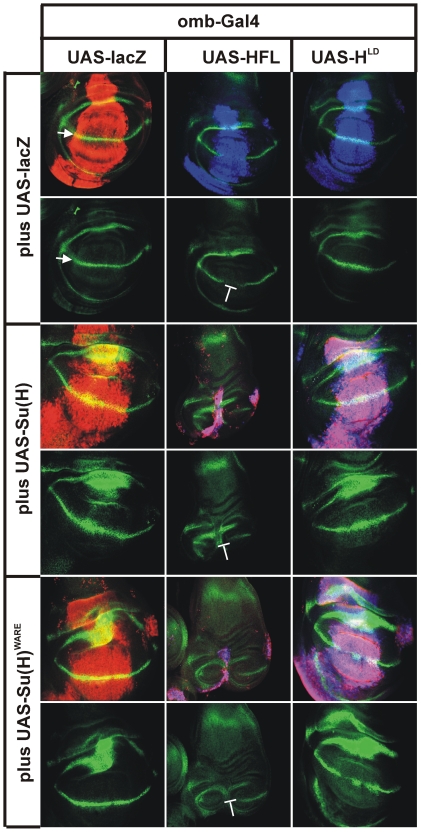
In vivo influence of the Su(H) and Hairless protein variants on the Notch target *wingless*. Wingless (wg) protein is expressed along the dorso-ventral boundary of the wing imaginal disc as a result of Notch signalling (see arrow in lac-Z control) [17], [18]. Wingless protein is shown in green in all panels. Su(H) and Hairless variants were overexpressed using the Gal4/UAS system within the central part of the wing disc (red in lac-Z control). Apart from the beta-galactosidase control, red depicts Su(H) protein. Hairless protein is shown in blue; overlap with Su(H) appears magenta and in addition with wg, it appears white. The control disc shows the expression domain (red, beta-galactosidase) of the *omb*-Gal4 driver. Wingless (Wg) protein outlines the wing pouch and dissects it along the dorso-ventral boundary (arrow). The latter expression is induced by a Notch signal. Overexpression of wild type Hairless (blue) represses Wg expression (blunt bar). In contrast, the mutant H^LD^ is unable to repress Wg. Overexpression of wild type Su(H) (red) causes proliferation of the wing blade and a subtle expansion of Wg expression. A combined overexpression of Su(H) and Hairless gives a super-additive effect: the expression domain becomes very small and Wg expression is inhibited. The mutant H^LD^ has no such effect; the combination with Su(H) resemble the sole Su(H) overexpression reflecting lack of binding of the two proteins. Overexpression of mutant Su(H)^WARE^ results also in a slight overproliferation of the wing disc and subtle Wg expansion. Also in combination with Hairless, Su(H)^WARE^ strongly impedes proliferation and Wg expression. However, no such effect of H^LD^ on Su(H)^WARE^ can be observed.

First we analysed the consequences on the expression of the Notch target gene *wingless (wg)*. Wg is expressed in the developing wing imaginal disc in a ring outlining the presumptive wing pouch and along the dorso-ventral boundary, which eventually forms the margin of the wing (Fig. 5) [17], [18]. The constructs were induced singly or in combination in a central area of the wing disc. Overexpression of either Su(H) or Su(H)^WARE^ effected an overproliferation of the affected tissue, which is typical for Notch gain of function, suggesting that both caused the activation of Notch target genes. Accordingly, a subtle expansion of Wg expression was observed compared to the control (Fig. 5). The ectopic Wg expression in the inner and outer rings was not anticipated since *wg*, according to several publications, is not a Notch target in this part of the tissue [19]–[21]. As expected, overexpression of Hairless HFL antagonized the expression of Wg at the intersection of the HFL expression domain and the presumptive margin and led to less tissue due to cell death. However, induction of the mutant H^LD^ was indistinguishable from wild type confirming complete loss of Su(H) binding in this mutant.

A combined overexpression of Hairless and Su(H) led to a remarkable loss of tissue and repression of *wg* expression (Fig. 5), which is in accordance with earlier observations and can be explained by the formation of a large surplus of repressor complexes formed [10]–[12], [22]. The mutant H^LD^ fails to bind to Su(H), therefore, the combined overexpression resembled the phenotype of the sole Su(H) overexpression (Fig. 5). Again, in combination with HFL, Su(H)^WARE^ behaved largely identical as wild type Su(H), indicating the normal formation of repressor complexes (Fig. 5), which also confirms our S2 cell culture results.

### Activity Su(H) and Hairless protein variants during eye development of the fly

**Figure 6:**
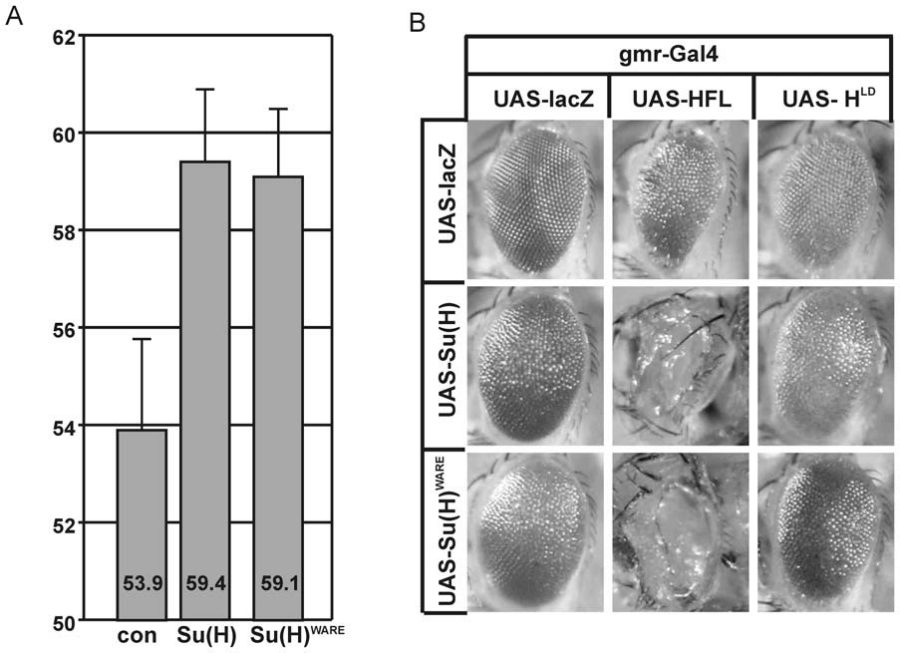
Overexpression of Su(H) und Hairless protein variants affect *Drosophila* eye development. A) Ectopic expression of Su(H) (lane 2) and Su(H)^WARE^ (lane 3) cause an increase of eye size compared with a control lac-Z (con, lane 1) ectopic expression using the gmr-Gal4 driver line. Eye size of male flies was measured from 19 to 20 individuals of each genotype. Average area are given in kilo pixel (kpx). Error bar represents standard deviation. B) The UAS transgenes Su(H) and Su(H)^WARE^ were expressed singly or in combination together with full length Hairless HFL or H^LD^, respectively, using gmr-Gal4 as driver line. As a control, lacZ was overexpressed which gives a wild type looking eye. Su(H) and Su(H)^WARE^ induce a slight overgrowth of tissue resulting in enlarged eyes. In contrast, eye specific overexpression of HFL causes smaller eyes with irregular arrangement of the ommatidia, giving a rough appearance. Overexpression of H^LD^ causes a nearly wild type eye. A combination of Su(H) with HFL results in a complete loss of the ommatidia: only a small eye slit remains that is totally smooth and devoid of any red eye pigment. In contrast a combined overexpression of Su(H) and H^LD^ causes enlarged eyes similar to the sole Su(H) overexpression, demonstrating lack of protein binding. Overexpression of Su(H)^WARE^ alone or in combination with Hairless variants gives similar results suggesting again that the WARE mutation does not influence repressor complex formation.

To substantiate these results we extended our analysis to the *Drosophila* eye, where Notch signalling is required at multiple, subsequent steps (reviewed in [23]). We used the gmr-Gal4 line that drives expression in the differentiating retina [24]. As reported earlier [25], overexpression of Su(H) is characterized by an overproliferation of eye tissue, as expected for a gain of Notch activity (Fig. 6). A likewise phenotype was induced by the overexpression of Su(H)^WARE^ in accordance with its ability to assemble an activator complex together with Notch (Fig. 6a). In contrast, expression of the antagonist Hairless resulted in small, irregular eyes by interference with several Notch dependent processes and subsequent induction of apoptosis (Fig. 6b) [26]–[28]. In combination, Su(H) and Hairless overexpression led to almost eyeless flies: only small slits remained lacking ommatidial structures or eye color (Fig. 6b). Again, a similar result was observed with Su(H)^WARE^ indicating little differences compared to wild type Su(H). In contrast, overexpression of the mutant H^LD^ had little biological effect and did not influence the activity of the Su(H) constructs (Fig. 6b). Similar results were observed in the process of lateral inhibition during bristle formation on the thorax ([10] and not shown).

**Figure 7:**
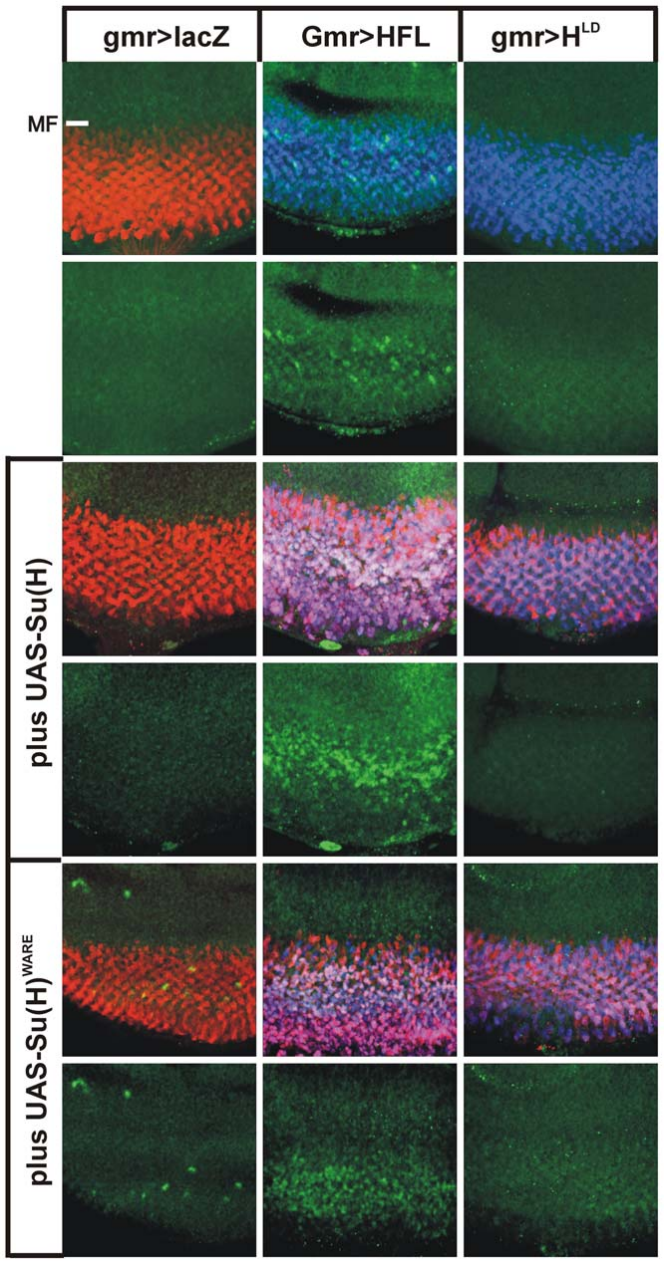
Regulation of apoptosis by Su(H) and Hairless variants during *Drosophila* eye development. The indicated UAS transgenes were overexpressed alone or in combination using the gmr-Gal4 driver line. As control the UAS-lacZ gene was likewise overexpressed. Gmr-Gal4 is active in the developing photoreceptor cells that arise posterior to the morphogenetic furrow (MF). Overexpression was visualized with specific antibodies against beta-galacotsidase (red, control), Su(H) (red) and Hairless (blue). In the merge, expression appears magenta. Apototic cells were detected with an antibody directed against activated Caspase 3 (green). A surplus of Hairless induces apoptosis which is strongly enhanced by the combined overexpression with either Su(H) or Su(H)^WARE^. In contrast, H^LD^ had no such an impact.

The small eyes resultant from Hairless overexpression are partly due to apoptosis induced by the repression of several Notch target genes and the concomitant downregulation of EGFR signalling activity [27]–[29]. We wondered whether the primary cause of the extreme adult phenotypes seen with the combined overexpression of HFL and Su(H) was also due to apoptosis. This was confirmed by staining for the cleaved, active form of Caspase-3 – the final effector Caspase in the apoptotic cascade [30] – which was dramatically increased in the eye discs of the relevant combinations (Fig. 7). In contrast no apoptosis was seen upon ectopic expression of the mutant H^LD^ (Fig. 7). This result is in agreement with the adult eye phenotype. Because H^LD^ fails to bind to Su(H), H^LD^ cannot be recruited to the respective promoters to assemble the respective repression complex, explaining the absence of apoptosis.

## Discussion

While we have detailed knowledge of the structure of the ternary activator complex (CSL-ICN-MAM), repression of Notch signal transduction is less well understood (overview in [4], [7]). We have started a more detailed analysis on the Notch repression complex in *Drosophila* which contains the CSL-type DNA binding protein Su(H), the bridging platform protein Hairless and the two general corepressors, Groucho and C-terminal binding protein [9], [11], [12], [31]. We have shown recently that Su(H) and Hairless form a high affinity complex that involves the CTD of Su(H) and the NT-domain of Hairless [10]. Moreover, our work demonstrated that Notch can outcompete Hairless for the binding of Su(H). This observation is startling for two reasons: firstly, both Notch and Hairless show comparable affinity for Su(H) which is in the nanomolar range, and secondly, the two molecules contact different sites in Su(H), excluding a simple competition scenario [10]. Presumably, the switch between activator and repressor status is more complicated and may involve structural changes in Su(H) [32], [33].

In order to provide the molecular basis for a deeper understanding of these processes we have started to map the Su(H)-Hairless sites of interaction. In Hairless, we have been able to determine a single amino acid that is crucial for the binding of Su(H) without overtly disturbing Hairless structure [10]. Here we identify two residues W491 and R493 in the CTD of Su(H) that likely contribute to the binding of Hairless based on our yeast two-hybrid data. Strikingly, the WARE mutant behaved similar to wild type in our cellular and in vivo assays. How could this discrepancy in our results be resolved? The simplest explanation is the involvement of one or more additional contact sites in Su(H) located elsewhere that sufficiently stabilize the binding of the full length Hairless and Su(H) proteins in vivo, but not the interaction between CTD and Hairless in the yeast assay. As we have already mutated most of the surface exposed residues in Su(H) CTD without affecting Hairless binding, we must conclude that single mutations are not disruptive and that we have not fortuitously hit upon the right combination of multiple amino acids in Su(H) to completely disrupt binding. Certainly the determination of the Su(H)-Hairless complex crystal structure will clarify the role of these residues in Hairless binding, as well as define other important interaction regions.

**Figure 8:**
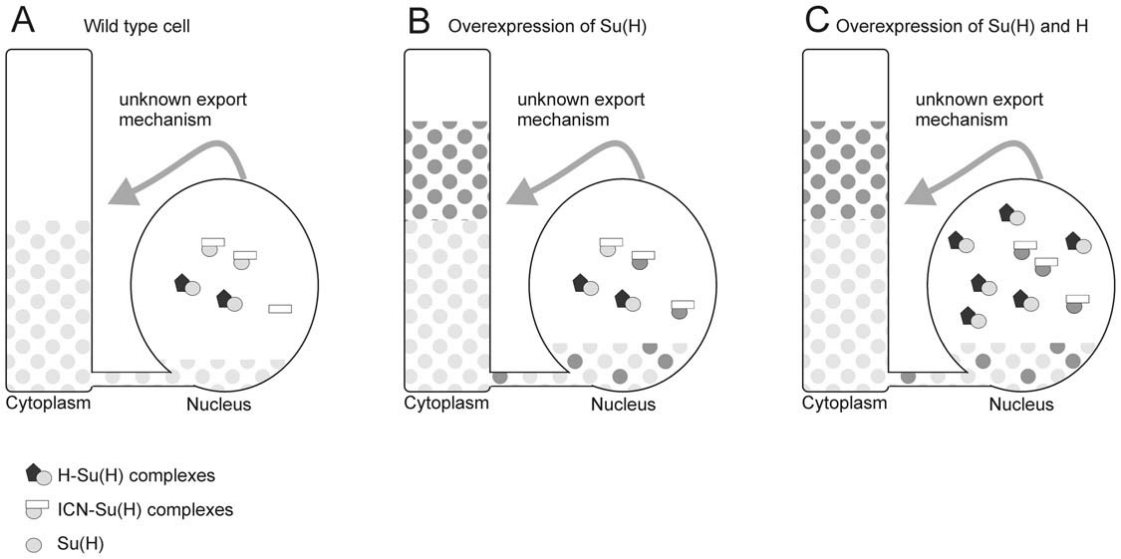
Modell of a Su(H) cytoplasmic pool. A) The large cytoplasmic pool of Su(H) is not available for complex formation in the nucleus, however, is used to replenish nuclear Su(H). This explains the apparent limitation of Su(H) when ICN is overexpressed. The export mechanism is currently unknown and may represent a further level of Su(H) regulation. B) Overexpression of Su(H) increases the cytoplasmic protein pool, thereby raising the level of available nuclear Su(H). This may cause Notch gain of function phenotypes in tissues, where Notch signalling takes place, or in the presence of exogenous ICN. The darker colour represents mutant Su(H) protein to exemplify the model; wild type Su(H) would behave identical. C) Combined overexpression of Su(H) and Hairless allows formation of repressor complex, as long as endogenous wild type Su(H) is available from the cytoplasmic pool. Lack of binding of Hairless to mutant Su(H) predicts an enrichment of activator complexes containing the mutant Su(H) and ICN.

However, we also need to consider the quantitative differences in the approaches. In the yeast the molecules are tested in a near 1∶1 molar ratio (assuming equal expression and stability of the proteins), whereas both in vivo approaches were based on overexpression and hence assayed with an excess of Su(H). S2 cells lack Notch but express both Su(H) and Hairless [13], [34], [35]; and the endogenous levels of Su(H) are sufficient for a strong response to experimental ICN doses [13], [14], [36]. Addition of Su(H) enhances the ICN response nearly fourfold [10], [14], [36], similar to the in vivo situation, where overexpression of Su(H) elicits Notch gain of function phenotypes [12], [22], [37]. We have no information on the amount of Su(H) in a cell that is freely available for binding to either Notch or Hairless, and the above observations suggest that Su(H) is limiting. Clearly, Su(H) occupancy on Notch target gene promoters is highly dynamic and enhanced by the presence of ICN [35]. However, *Drosophila* cells express high levels of Su(H) in the cytoplasm which is rather unconventional for a transcription factor [22], [38], [39]. The mechanisms underlying Su(H) nuclear import/export are little understood and may involve Notch signalling and repression, respectively [38]–[41]. Most likely cytoplasmic Su(H) is unavailable for transcriptional complex formation, be it repressor or activator complex. Moreover, Su(H) from the cytoplasm may resupply the nucleus once Su(H) is bound in complexes such that a steady level of free nuclear Su(H) is achieved. Overexpression of Su(H) may raise this level considerably, explaining the increase of Notch output in the cell culture as well as in fly tissue. Hairless would access the same free pool of Su(H), however, only bind to wild type protein to build up repressor complexes and silence Notch target genes, whereas Notch could access both mutant and wild type Su(H). Assuming a large enough pool of wild type Su(H), then repressor complex formation might in fact be as efficient in the presence of mutant as of wild type overexpressed Su(H) (Fig. 8). This rather speculative model would be in agreement with an in vivo reduced or lack of binding between Hairless and Su(H)^WARE^ and concomitant repression by a complex consisting primarily of Hairless and endogenous Su(H). Experiments addressing the exact composition of the repressor complexes, i.e. the presence of mutant Su(H), may help support this model.

## Materials and Methods

### Yeast two-hybrid experiments

Single, double or triple missense mutations in CTD were introduced using the QuickChange^R^ II XL site-directed mutagenesis kit (Stratagene). All mutants were sequence verified (StarSeq, Mainz). CTD mutants were cloned into pJG vector [42] and tested for protein interactions with pEG constructs as described previously [10]. Primer sequences are available upon request. The yeast three-hybrid experiments were performed with the N-ANK domain cloned in the pESC-Leu vector (Stratagene) and pEG-MamN as outlined in [10].

The CTD^WARE^ mutant DNA was excised with *Msc* I and *Eco* 52I from the CTD^WARE^ pJG-construct and reintroduced in likewise digested Su(H) cDNA to generate the mutant full length construct Su(H)^WARE^. It was shuttled into pRmHa-3 [43] and pUAST-attB- vectors [15] for subsequent in vivo analyses.

### Electro-mobility shift assays - EMSA

DNA binding assays of Su(H) and Su(H) mutants were performed according to standard protocols using a double stranded DNA-oligomer (made by hybridization of primers 5′ GGT TCT TTC AGC TCG GTT CCC ACG CCA CGA GCC AC 3′ and 5′ TTG GGT GGC TCG TGG CGT GGG AAC CGA GCT GAA AG 3′ and labelled with Klenow polymerase) containing the *E(spl)*m8-S1 Su(H) binding site [44] and Su(H) proteins produced from cDNA by in vitro transcription/translation using the TNT®Coupled Reticulocyte Lysate System (Promega).

### Cell culture assays

For cell culture experiments *Drosophila* Schneider S2 cells, obtained from the *Drosophila Genomics Resource Centre* DGRC (Indiana University, Bloomington USA), were transfected with the respective constructs and the activity measured with a Notch responsive luciferase reporter (NRE-reporter) as described previously [13]. Renilla expression plasmid (tk-Renilla; Promega) was cotransfected as internal control. Reporter activation elicited by transfection with pMT-ICN was taken as 100% [45]. Cotransfection with Su(H) and Hairless constructs were analysed as described before [10]. CuSO_4_ was used to induce protein expression 6 h after transfection. Luciferase activity was measured 18 h later in duplicate (Lumat LB 9507, EG & Salem, MA) using the dual-luciferase reporter assay system (Promega).

### Analysis of mutant Hairless and Su(H) transgenes in vivo

Transgenic Su(H)^WARE^ flies were generated with the PhiC31 integrase-based integration system [15] to avoid position effects and allow for a direct comparison with likewise integrated wild type Su(H) [10]. For co-overexpression experiments, the Su(H)^WARE^ insertion located at 96E was recombined with HFL and H^LD^, respectively, located at 68E [10]. Tissue specific overexpression was achieved with the Gal4/UAS-system [16] using omb-Gal4 and gmr-Gal4 driver lines (http://flybase.org).

Antibody staining of imaginal discs was performed as described before using the following antisera: anti H-A [46], anti-Su(H) (Santa Cruz Biotech), anti-cleaved Caspase 3 (NEB Cell Signaling Technologie) anti-wg as well as anti-beta-galactosidase (developed by M. Cohen and J.R. Sanes, respectively, and obtained from Developmental Studies Hybridoma Bank developed under the auspices of the NICHD and maintained by The University of Iowa, Department of Biology, Iowa City, IA 52242). Secondary antibodies coupled to DATF, Cy3 or Cy5 were purchased from Jackson Laboratory (Dianova). Samples were mounted in Vectashield (Vector Lab) and analyzed on a Zeiss Axiophot linked to a Bio-Rad MRC1024 confocal microscope. Flies were monitored using an ES120 camera (Optronics), with Pixera Viewfinder Version 2.0 software. Pictures were assembled with Corel-PhotoPaint and CorelDRAW Version 9.0 software.
